# Identification of Hybrid *Indica* Paddy Rice Grain Varieties Based on Hyperspectral Imaging and Deep Learning

**DOI:** 10.3390/bios15100647

**Published:** 2025-09-30

**Authors:** Meng Zhang, Peng Li, Wei Dong, Shuqi Tang, Yan Wang, Runmei Li, Shucun Ju, Bolun Guan, Jingbo Zhu, Juanjuan Kong, Liping Zhang

**Affiliations:** 1 Agricultural Economy and Information Research Institute, Anhui Academy of Agricultural Sciences, Hefei 230031, China; zhangmeng@aaas.org.cn (M.Z.);; 2 College of Engineering, South China Agricultural University, Guangzhou 510642, China; 3 School of Computer and Information Engineering, Fuyang Normal University, Fuyang 236037, China; 4 Anhui Disater Earnly Warning and Agricultural Meteorological Information Center, Hefei 230031, China

**Keywords:** paddy rice grain, variety classification, hybrid *indica*, hyperspectral imaging, machine learning, interpretability analysis

## Abstract

Paddy rice grain variety classification is essential for quality control, as different rice varieties exhibit significant variations in quality attributes, affecting both food security and market value. The integration of hyperspectral imaging with machine learning presents a promising approach for precise classification, though challenges remain in managing the high dimensionality and variability of spectral data, along with the need for model interpretability. To address these challenges, this study employs a CNN-Transformer model that incorporates Standard Normal Variate (SNV) preprocessing, Competitive Adaptive Reweighted Sampling (CARS) for feature wavelength selection, and interpretability analysis to optimize the classification of hybrid *indica* paddy rice grain varieties. The results show that the CNN-Transformer model outperforms baseline models, achieving an accuracy of 95.33% and an F1-score of 95.40%. Interpretability analysis reveals that the model’s ability to learn from key wavelength features is significantly stronger than that of the comparison models. The key spectral bands identified for hybrid *indica* paddy rice grain variety classification lie within the 400–440 nm, 580–700 nm, and 880–960 nm ranges. This study demonstrates the potential of hyperspectral imaging combined with machine learning to advance rice variety classification, providing a powerful and interpretable tool for automated rice quality control in agricultural practices.

## 1. Introduction

Rice, a critical global staple crop, plays a central role in food security and the quality of life, especially for populations in Asia, where its yield and quality are of paramount importance. *Indica* rice, one of the main subspecies, dominates in cultivation area due to its remarkable adaptability, high yield, and short growth cycle [1]. The rise of hybrid rice technology has substantially boosted rice productivity and resilience, playing a key role in narrowing the food supply-demand gap in numerous nations [2]. However, the rapid progress in hybrid breeding has resulted in the emergence of numerous *indica* rice varieties, creating new challenges for variety management and market regulation. Despite their morphological similarities, these varieties differ markedly in internal quality, taste, and market prices [3]. Consequently, establishing an accurate and efficient method to identify *indica* paddy rice grain varieties is crucial for ensuring rice quality, regulating market stability, and enhancing traceability management.

Rice variety identification currently depends mainly on traditional methods, including expert judgment, morphological trait comparison, and molecular markers [4]. While these methods help identify rice varieties to some extent, they are hindered by low efficiency, high costs, and significant subjectivity, rendering them inadequate for the high-throughput and automated detection needs of modern agriculture. Recent advancements in information acquisition and intelligent sensing technologies have led to the widespread application of various non-destructive testing techniques, such as image recognition, hyperspectral imaging, Raman spectroscopy, terahertz imaging, and X-ray radiography [5,6,7,8], in agricultural product quality assessment. Among these emerging technologies, hyperspectral imaging is particularly notable for its high resolution and broad spectral coverage [9], allowing continuous spectral data acquisition from grain samples across a wide range of wavelengths. This capability enables the detection of chemical composition differences, making hyperspectral technology highly sensitive and precise for rice variety identification.

In recent years, the integration of hyperspectral imaging with machine learning techniques has yielded promising results in rice variety identification. Yang et al. [10] developed multiple classification models for identifying five rice varieties, with the Partial Least Squares Discriminant Analysis (PLS-DA) model achieving a classification accuracy of 95%. Similarly, Kang et al. [11] gathered hyperspectral data within the 475–1000 nm range from five visually similar rice varieties. They preprocessed the data with First Derivative (FD) for denoising and T-Distributed Stochastic Neighbor Embedding (T-SNE) for dimensionality reduction, achieving a classification accuracy of 90.6% using PLS-DA on the test set. Sun et al. [12] collected hyperspectral data from five rice varieties and applied Bootstrapping Soft Shrinkage (BOSS) for spectral data dimensionality reduction. They subsequently built a Support Vector Machine (SVM) classification model, achieving a recognition accuracy of 91.48% on the test set. In a separate study, Ge et al. [13] developed a hyperspectral rice dataset comprising six varieties, extracting average spectra from the Region of Interest (ROI) covering the entire grain, and achieved a classification accuracy of 94.92% using SVM. These studies underscore the effectiveness of machine learning algorithms, such as PLS-DA and SVM, for rice variety classification. However, traditional machine learning methods often struggle with high-dimensional and complex hyperspectral data, failing to fully capture the intricate features within the data. Their capacity to generalize to unseen data is often limited, and they may fail to capture non-linear relationships within the data [14]. Furthermore, as the dataset size grows, these methods may encounter reduced computational efficiency and scalability. In contrast, deep learning methods are more adept at learning complex, hierarchical features from large datasets. For example, Qiu et al. [15] used Wavelet Transform (WT) to preprocess hyperspectral data from four rice varieties and compared the performance of K-Nearest Neighbor (KNN), SVM, and Convolutional Neural Network (CNN) models. The results revealed that the CNN model outperformed both KNN and SVM, achieving superior classification accuracy. Similarly, Yu et al. [16] introduced a lightweight CNN for classifying 19 rice varieties with varying flavors. The proposed model achieved a classification accuracy of 98.98%, underscoring the capacity of deep learning models to handle complex, high-dimensional data more effectively than traditional methods.

While previous studies have made valuable contributions to paddy rice grain variety identification through hyperspectral data, they often overlook key factors such as geographic origin, harvest time, and field conditions, which can substantially influence the reliability of classification outcomes. Although CNNs have shown promise in processing complex hyperspectral data, they are limited in capturing long-range dependencies within the spectral data [17], which are crucial for accurate classification. Furthermore, existing models lack interpretability [18], complicating the understanding of which specific spectral bands or grain components contribute most to classification decisions, thereby limiting the model’s practical applicability and transparency.

This study integrates and refines existing methods to enhance the performance of paddy rice grain variety classification models, while also improving their reliability and interpretability. Specifically, (1) the study focused on hybrid *indica* rice with similar parental lines and uniform cultivation conditions to minimize the uncertainty introduced by external factors. (2) A CNN-Transformer model was employed to capture both local and global spectral features, addressing CNN limitations in long-range dependencies and enhancing overall model performance. (3) Shapley Additive Explanation (SHAP) was applied to evaluate model interpretability, identifying key spectral wavelengths and grain components contributing to classification decisions.

## 2. Materials and Methods

### 2.1. Sample Preparation

This study selected 13 *indica* rice varieties as experimental subjects, including JLY1212, XLYX128, TLY1332, LJY1212, GLYYNSM, HY82, YXLYFXZ, HXYYZ, HLY898, QGY822, QY822, QZY836, and YLY506. All selected rice varieties were hybrids, with certain parental lines being similar. To minimize interference from factors such as water, fertilizer, and environmental conditions, rice was uniformly planted and harvested between March and August 2023 in He Ping Village, Huoqiu County, Anhui Province, China (116°3′ E, 23°4′ N), with consistent field management practices throughout the growth period. Sowing took place in mid-March under plastic arch shelters for seedling cultivation. In mid-April, rice seedlings were manually transplanted, with water and fertilizer management following local farming practices. Mechanical harvesting was performed at the wax-ripe stage, defined by yellow panicles, well-filled grains, and approximately 85% of kernels appearing semi-translucent. After harvesting, the rice grains were arranged in storage boxes in a 4 × 7 pattern by variety to facilitate subsequent hyperspectral data collection. In total, 130 storage boxes were prepared for the experiment, with 10 boxes allocated to each rice variety. To ensure an independent evaluation, for each rice variety, two boxes were randomly selected and reserved as the independent test set prior to model development, and the remaining eight boxes per variety were used for training.

### 2.2. Hyperspectral Imaging System and Spectral Acquisition

#### 2.2.1. Hyperspectral Imaging System

The hyperspectral imaging system employed in this experiment consists of a visible and near-infrared hyperspectral camera (Specim FX10, Specim, Spectral Imaging Ltd., Oulu, Finland), 280W halogen lamps (DECOSTAR 51S, Osram Corp., Munich, Germany), and a mobile platform (HXY-OFX01, Red Star Yang Technology Corp., Wuhan, China). The system was operated via the Lumo-Scanner 4.8 (Specim, Spectral Imaging Ltd., Oulu, Finland). All data collection was performed in a darkroom environment to minimize ambient light interference. The sample was positioned 32 cm from the camera lens to ensure optimal image acquisition conditions. The system was calibrated before data collection to ensure the precision and accuracy of the acquired spectra. Table 1 provides the detailed specifications of the system.

#### 2.2.2. Spectral Data Extraction

To reduce the noise caused by uneven light intensity distribution, the acquired images *I*_0_ were calibrated. Calibration was performed using images of the standard white reference board *I_W_* and the black reference image *I_B_* obtained by covering the lens [19]. The corrected image *I* was calculated using the following equation:

(1) $$I=\frac{I_{0}-I_{B}}{I_{W}-I_{B}}$$

**Figure 1:**
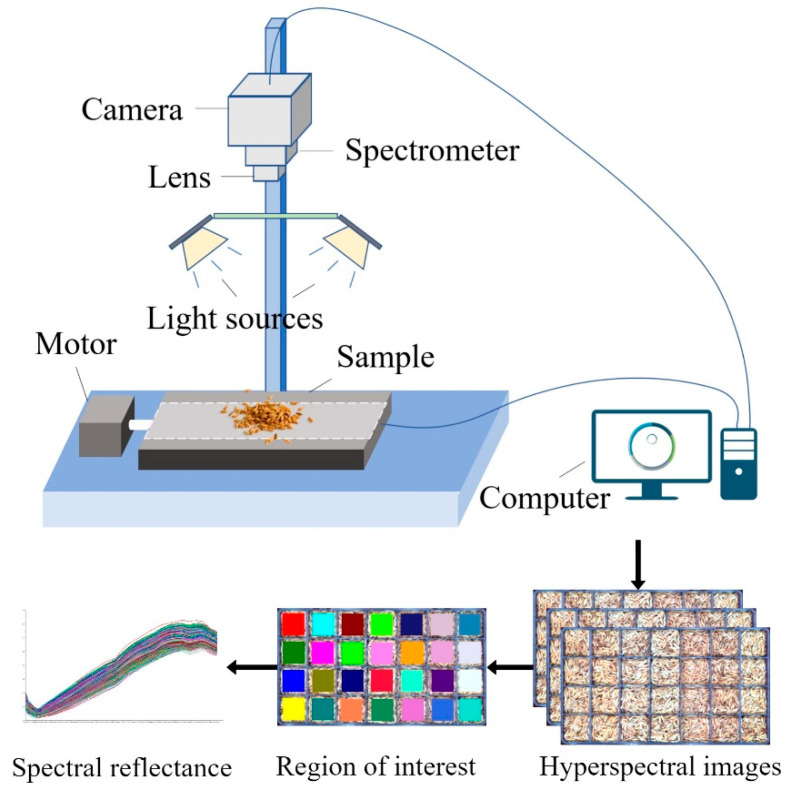
Data acquisition process.

After spectral data collection, a ROI was selected within each storage compartment of the boxes. A centered 50 × 50-pixel square ROI was defined within each grid cell, and the mean spectrum of all ROI pixels was used as the representative spectrum for that pile. One spectrum was therefore obtained per grid cell, yielding 3640 spectra in total. Data partitioning adhered to the predefined box-level split, resulting in 2912 training samples and 728 independent test samples. The hyperspectral equipment and data acquisition process are shown in Figure 1.

### 2.3. Principal Component Analysis

Principal Component Analysis (PCA) is a technique that projects high-dimensional data into a new coordinate system, simplifying the visualization of complex datasets in lower dimensions. PCA identifies the directions that capture the most variance by projecting the data onto principal components, facilitating the separation and identification of patterns [20]. In hyperspectral data analysis, PCA is especially useful for visualizing relationships and clusters between different wavelengths, uncovering underlying structures that may be obscured in the original high-dimensional space. This provides insights into data distribution and aids further analysis, such as classification and feature selection.

### 2.4. Spectral Preprocessing Methods

Data preprocessing plays a critical role in hyperspectral data analysis by enhancing the accuracy and robustness of classification models. Hyperspectral data are often contaminated by various types of noise, such as instrumental noise, baseline drift, and scattering effects, which can obscure meaningful spectral information [21]. To address these issues, this study employs five preprocessing techniques: Savitzky–Golay (SG) smoothing to reduce high-frequency noise, Baseline Correction (BC) to eliminate baseline drift, Standard Normalized Variate (SNV) transformation to correct scattering effects, De-trending (DT) to remove long-term trends and systematic errors, and FD to enhance subtle spectral variations. These techniques were chosen to address different noise sources and distortions in the data, aiming to identify the most effective preprocessing approach for rice variety classification. By comparing the performance of these techniques, the study identifies the most suitable approach for this task, thereby enhancing the reliability of the classification model.

### 2.5. Feature Extraction Methods

Feature wavelength selection is crucial in hyperspectral data analysis as it identifies the most informative wavelengths for classification, enhancing both model efficiency and accuracy. By concentrating on relevant bands, this approach reduces computational complexity and minimizes noise from irrelevant data [22]. This study employs four feature selection methods: Successive Projections Algorithm (SPA), which eliminates redundant bands through projection; Competitive Adaptive Reweighted Sampling (CARS), which adaptively selects the most significant wavelengths by weighing features; Uninformative Variable Elimination (UVE), which removes variables with minimal impact on reducing uncertainty; and Interval Variable Iterative Space Shrinkage Approach (IVISSA), which iteratively evaluates and selects the most important features. These methods were chosen for their distinct underlying principles, and the study aims to compare their effectiveness in selecting key wavelengths that most influence classification performance.

### 2.6. Modeling Algorithms

#### 2.6.1. Model Comparison Algorithms

In this study, PLS-DA, SVM, and One-Dimensional Convolutional Neural Network (1D-CNN) were selected for comparison to assess the performance of the newly proposed model. These algorithms are widely validated in hyperspectral data modeling and have demonstrated strong performance in various classification tasks. PLS-DA is a linear method commonly used in chemometrics for classification tasks. It projects high-dimensional data into a lower-dimensional space by identifying directions that best explain the covariance between input variables and target labels [23]. PLS-DA is particularly effective in handling collinearity in data, making it well-suited for hyperspectral data analysis. SVM, in contrast, is a powerful supervised learning algorithm that determines the optimal hyperplane by maximizing the margin between different classes [24]. It performs well with high-dimensional data, using kernel functions to map the input data to a higher-dimensional feature space, which enhances its robustness in classifying hyperspectral data, even with non-linearly separable classes.

The 1D-CNN employed in this study is a deep learning model tailored to process one-dimensional spectral data. The architecture includes three convolutional layers, each followed by Batch Normalization, ReLU activation, and MaxPooling [25]. The first convolutional layer applies eight filters with a kernel size of 5 and a stride of 1. The second and third convolutional layers use sixteen filters with a kernel size of 3 and a stride of 1. After the convolutional layers, the output is flattened and passed through a fully connected layer, followed by a linear output layer for classification. During training, Cross-entropy was used as the loss function, and the Adam optimizer was selected with a learning rate of 0.001. To prevent overfitting, a Dropout rate of 0.1 was applied. The batch size and epochs were set to 64 and 1000, respectively.

#### 2.6.2. CNN-Transformer

The proposed CNN-Transformer model integrates the strengths of both CNN and Transformer architectures to effectively process hyperspectral data for rice variety classification. The CNN component mirrors the 1D-CNN model described earlier, while the Transformer component is introduced to enhance the model’s ability to capture long-range dependencies in the hyperspectral data. The Transformer encoder utilizes a self-attention mechanism to capture relationships between different spectral bands, enhancing the model’s ability to understand complex dependencies across wavelengths [26].

In the self-attention mechanism, input features are transformed into three distinct matrices: the query matrix *Q*, the key matrix *K*, and the value matrix *V*, which represent the spectral data features. Attention scores are computed by measuring the similarity between each query vector and all key vectors [27], which are then normalized using the softmax function to convert the similarities into probability distributions:

(2) $$Attention(Q,K,V)=softmax\left(\frac{QK^{T}}{\sqrt{d_{k}}}\right)V$$

In this mechanism, the dot product of *Q* and *K* computes the similarity, and the softmax function ensures that the resulting weights emphasize the most relevant spectral features. By applying these attention weights to the value matrix *V*, the model learns to prioritize important spectral bands while reducing the impact of less relevant ones. This approach enables the model to capture global relationships within the spectral data [28], which is especially beneficial for hyperspectral image classification, where spectral features may display complex interactions.

**Figure 2:**
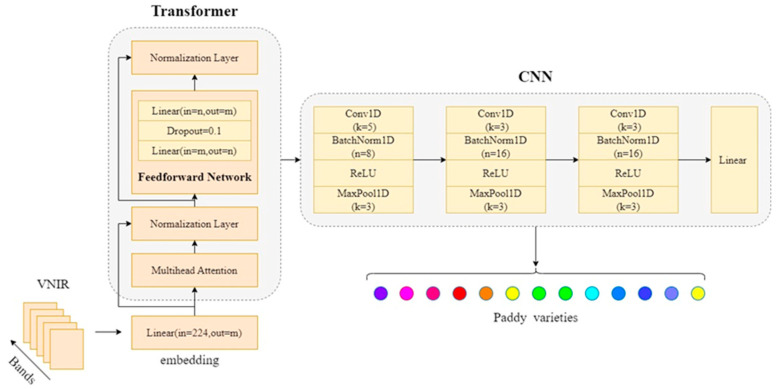
CNN-Transformer model structure.

After the Transformer encoder extracts global features, the CNN component refines them by capturing local spatial patterns. The architecture of this model mirrors the 1D-CNN model described earlier, consisting of three convolutional layers followed by batch normalization, ReLU activation, and MaxPooling. The same filter sizes, kernel sizes, strides, and pooling operations are applied to extract features from the input data. After processing through both CNN and Transformer components, the model flattens the output and passes it through a fully connected layer before the final classification layer, which outputs the predicted class probabilities. The model is trained using the same parameters as the 1D-CNN model, ensuring consistency in the experimental setup. The structure of the CNN-Transformer model is shown in Figure 2.

### 2.7. Model Evaluation

To assess the performance of the models, several common classification metrics were used, including accuracy, precision, recall, and F1-score. Accuracy represents the proportion of correctly classified samples to the total number of samples, as defined in Equation (3). Precision measures the proportion of positive predictions that are correct, as defined in Equation (4). Recall quantifies the proportion of actual positives correctly identified by the model, as defined in Equation (5). The F1-score is the harmonic mean of precision and recall, offering a balance between the two metrics, as defined in Equation (6). Together, these metrics provide a comprehensive evaluation of the model’s classification performance, considering both overall accuracy and the ability to correctly identify positive and negative instances.

(3) $$\;\mathrm{Accuracy}=\frac{TP+TN}{TP+FN+FP+TN}$$

(4) $$\;\mathrm{Precision}=\frac{TP}{TP+FP}$$

(5) $$\;\mathrm{Pecall}=\frac{TP}{TP+FP}$$

(6) $$\mathrm{Fl}-\mathrm{Score}=\frac{2^{*}Precision\times Recall}{Precision+Recall}$$

where True Positive (*TP*) refers to instances correctly predicted as positive, True Negative (*TN*) represents those correctly predicted as negative, False Positive (*FP*) denotes instances incorrectly predicted as positive, and False Negative (*FN*) refers to those incorrectly predicted as negative.

### 2.8. Interpretability Method

To interpret and explain the predictions of machine learning models, SHAP was employed in this study. SHAP is a game-theory-based method that attributes each feature’s contribution to a model’s output by calculating the average marginal contribution of each feature across all possible combinations of features [29]. It provides a unified measure of feature importance and interactions, enabling a clearer understanding of how individual features influence model predictions. By assigning a SHAP value to each feature, it becomes possible to determine not only the significance of each feature but also the direction and magnitude of its effect on the model’s output [30].

In this study, SHAP was used to visualize the contribution of each spectral band to the model’s predictions. This enables the identification of the most important wavelengths for classification tasks. Moreover, different spectral bands exhibit distinct responses to rice grain composition, and SHAP values aid in interpreting how specific wavelengths correspond to different rice varieties. Analyzing these contributions provides insights into how spectral data related to rice grain components influence the model’s ability to distinguish between varieties, offering a deeper understanding of the relationships between spectral features and rice variety classification.

### 2.9. Software

In this study, ENVI 5.2 was used to extract spectral data from the hyperspectral images, while The Unscrambler X 10.4 facilitated spectral preprocessing. MATLAB 2024a was used to select the most relevant wavelengths for model input. For machine learning modeling and explainability analysis, Python 3.9 and PyTorch 1.10 were used, leveraging their robust libraries for data processing and model interpretation.

## 3. Results and Discussion

### 3.1. Original Spectral Analysis

**Figure 3:**
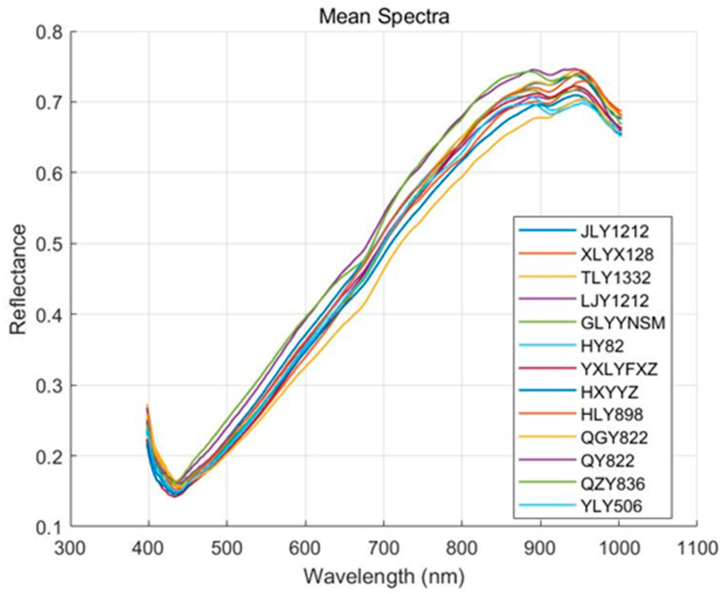
The average spectral reflectance of different grains of rice varieties.

Figure 3 illustrates the mean spectral reflectance of 13 rice varieties across the 397–1003 nm wavelength range. The spectral curves of all rice varieties display similar trends, with distinct features observed in specific wavelength regions. In the blue light range (400–500 nm), reflectance is relatively low, likely due to strong absorption by surface compounds such as flavonoids, anthocyanins, and other phenolic compounds in the rice husk [31]. In the green light region (500–600 nm), reflectance slightly increases, likely due to reduced pigment absorption and enhanced surface scattering. In the 600–800 nm region, reflectance increases rapidly, showing a characteristic “red edge” feature. This sharp rise in reflectance is linked to changes in the internal structure of the rice grain and increased scattering of near-infrared light. In the near-infrared region (800–1000 nm), reflectance stabilizes, reflecting weak absorption by internal components such as starch and proteins, along with strong scattering from the grain structure [32]. Around 930 nm, some varieties show a slight decrease in reflectance, corresponding to water absorption features in the grain, a typical characteristic in the near-infrared region [33]. While the spectral curves are highly similar across varieties, subtle differences exist in certain features, such as the green light peak around 550 nm, variations in the red edge around 750 nm, near-infrared plateau reflectance between 800 and 950 nm, and absorption depth near 970 nm. These differences may result from variations in husk pigment composition, grain density, chemical composition, and moisture content [34], offering useful insights for subsequent feature selection and classification modeling.

### 3.2. PCA Explanatory

**Figure 4:**
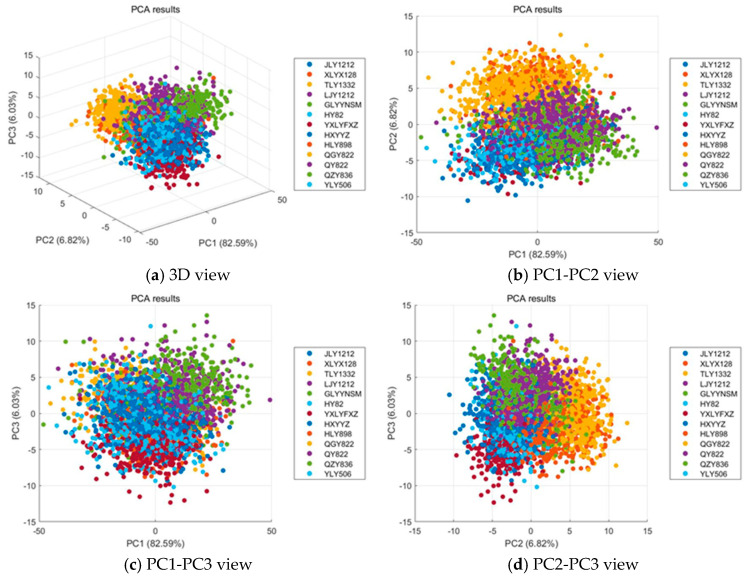
PCA results of hyperspectral data for rice varieties from different perspectives: (**a**) 3D view; (**b**) PC1-PC2 view; (**c**) PC1-PC3 view; (**d**) PC2-PC3 view.

Figure 4 illustrates the results of PCA applied to the hyperspectral data of 13 rice varieties. The first three principal components (PC1, PC2, and PC3) explain a cumulative variance of 95.44%, with PC1 accounting for 82.59%, PC2 for 6.82%, and PC3 for 6.03%. As shown in Figure 4a, all samples merge into one large, dense cloud with no clear global separation, while each variety still forms small local subclusters; the distribution is elongated along PC1 and the boundaries between varieties remain porous. The relationships between different principal components are further explored in the 2D plots. As shown in Figure 4b, the PC1–PC2 view accounts for 89.41% of the variance and arranges the data into partially ordered bands along PC1, yet substantial intermixing persists across PC2 and heavy overlaps appear near the center of the manifold. As shown in Figure 4c, adding PC3 introduces limited additional dispersion that reflects weak secondary differences without forming distinct groups. As shown in Figure 4d, the PC2–PC3 projection explains only 12.85% of the variance and presents a largely mixed pattern with only mild peripheral aggregation, indicating that the remaining structure is too weak for simple linear separation. Overall, hyperspectral imaging effectively captures within variety structure that clusters into meaningful small groups, but extensive between variety overlap remains, so unsupervised linear projections provide limited separability and supervised representation learning is required to extract discriminative features for reliable rice variety classification.

### 3.3. Spectra Preprocession

**Figure 5:**
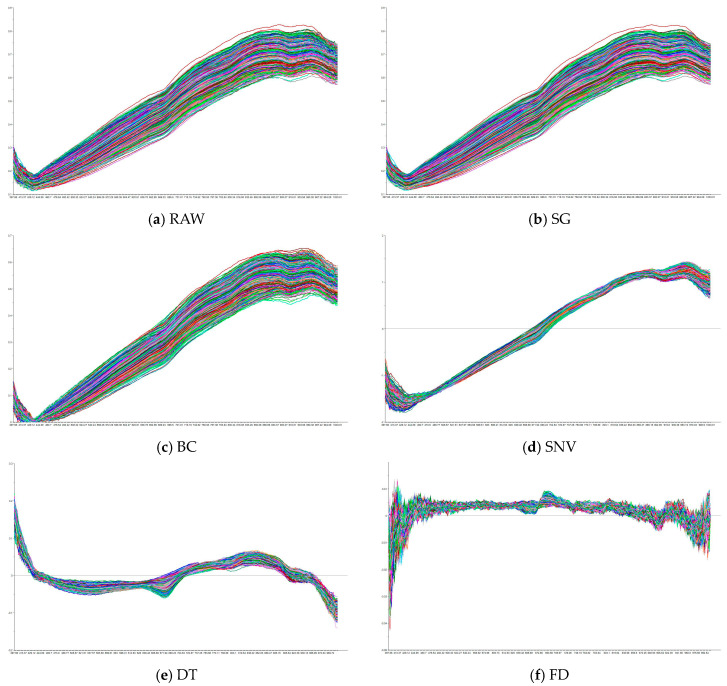
Spectral characteristic curves after different preprocessing methods: (**a**) RAW; (**b**) SG; (**c**) BC; (**d**) SNV; (**e**) DT and (**f**) FD.

Figure 5 illustrates the raw spectral curve alongside the spectra after applying different preprocessing methods. It can be observed that the raw spectral data exhibit significant noise and fluctuations, which are effectively mitigated through preprocessing. SG reduces high-frequency noise while preserving the overall trends and key spectral features, producing smoother and more stable spectra compared to the raw data. BC eliminates low-frequency drift, aligning the spectra and improving consistency across samples. SNV standardizes the spectra by removing scattering effects and normalizing baseline variations, though it introduces additional variability, accentuating differences between samples, especially in certain bands. DT removes long-term trends, emphasizing short-term fluctuations in the spectra, though it reduces overall smoothness, making the spectra appear more fragmented. Finally, FD enhances spectral details by emphasizing the rate of change, amplifying peaks and valleys; however, it also increases sensitivity to high-frequency noise, leading to more pronounced fluctuations. The differences between the raw and preprocessed spectra highlight how each method modifies the data to address noise while enhancing specific features, optimizing the data for further analysis.

### 3.4. Full Wavelength Modeling

The objective of this analysis is to identify the optimal combination of preprocessing methods and modeling algorithms for rice variety classification using hyperspectral data. Prior to training, 4-fold cross-validation was performed on the training set to select the best parameters and evaluate the model’s generalization ability. Table 2 presents the performance of four classification algorithms, each applied with five different preprocessing methods to the full spectral data.

The CNN-Transformer achieves the highest test accuracy (95.74%) and F1-score (95.79%) with the SNV preprocessing method, demonstrating its superiority in handling complex spectral data. 1D-CNN also demonstrates strong performance, particularly with FD, achieving a test accuracy of 94.64% and an F1-score of 94.73%, indicating that this model is particularly effective in capturing spectral features when paired with the appropriate preprocessing. SVM, with SNV preprocessing, shows significant improvements, with a test accuracy of 93.54% and an F1-score of 93.58%, highlighting its ability to produce robust results with well-preprocessed data. PLS-DA, while stable across different preprocessing methods, achieves lower performance than the other models, with a maximum test accuracy of 82.42% using SG preprocessing, indicating that its ability to capture spectral variance is less effective. Overall, the CNN-Transformer model consistently outperforms the other algorithms across most preprocessing methods, and this advantage is primarily architectural, with the Transformer branch capturing long-range spectral dependencies while the CNN branch contributes inductive biases such as local connectivity, weight sharing, and hierarchical receptive fields that extract stable local motifs and suppress noise. Moreover, compared with traditional machine learning approaches, the deep-learning framework learns features end-to-end and scales effectively to high-dimensional, large-sample hyperspectral datasets, collectively delivering higher accuracy, stronger generalization, and reduced dependence on preprocessing.

Moreover, the choice of preprocessing method plays a critical role in the model’s performance. SNV performs well across all algorithms, but it is not always the top-performing method. In PLS-DA and 1D-CNN, SNV leads to good performance but does not yield the highest accuracy. For instance, SG preprocessing outperforms SNV in PLS-DA regarding test accuracy (82.42%). Similarly, 1D-CNN exhibits slightly better performance with FD preprocessing (94.64% test accuracy) compared to SNV (93.96%). Nevertheless, SNV remains effective in normalizing the data and reducing noise, which enhances overall classification performance in these models. Interestingly, BC, DT, and FD preprocessing methods reduce the accuracy of the raw data in PLS-DA and SVM, possibly due to over-correction or the introduction of distortions in the spectral features [35]. In contrast, SG preprocessing results in slight performance degradation in 1D-CNN and CNN-Transformer, possibly due to excessive smoothing, which may blur finer spectral features essential for distinguishing between varieties [36]. These findings emphasize the importance of carefully selecting preprocessing methods to match the specific modeling algorithm, as inappropriate preprocessing can result in the loss of critical spectral information.

Notably, 1D-CNN may be susceptible to overfitting, particularly with RAW, SG, and BC preprocessing methods. The high training accuracy relative to the test accuracy suggests that the model may have been overfitted to the training data, limiting its generalizability. However, the CNN-Transformer model effectively mitigates this issue, demonstrating consistently high performance across all preprocessing methods without signs of overfitting. This suggests that CNN-Transformer is better equipped to generalize across different rice varieties, even with noisy or highly variable spectral data. In this task, SNV consistently performed well across all models, suggesting that the raw hyperspectral data contain significant scattering effects and baseline shifts. SVM showed a dramatic improvement in performance, particularly when combined with SNV preprocessing. The test accuracy of SVM increased substantially (from 88.73% to 93.54%), indicating that SVM has limited robustness against these spectral variations without preprocessing. The fact that SNV achieved good performance across all algorithms implies that the raw data contain considerable noise, with SNV playing a key role in mitigating this effect and improving the model’s ability to capture meaningful spectral features for classification.

### 3.5. Feature Wavelengths Distributions

**Figure 6:**
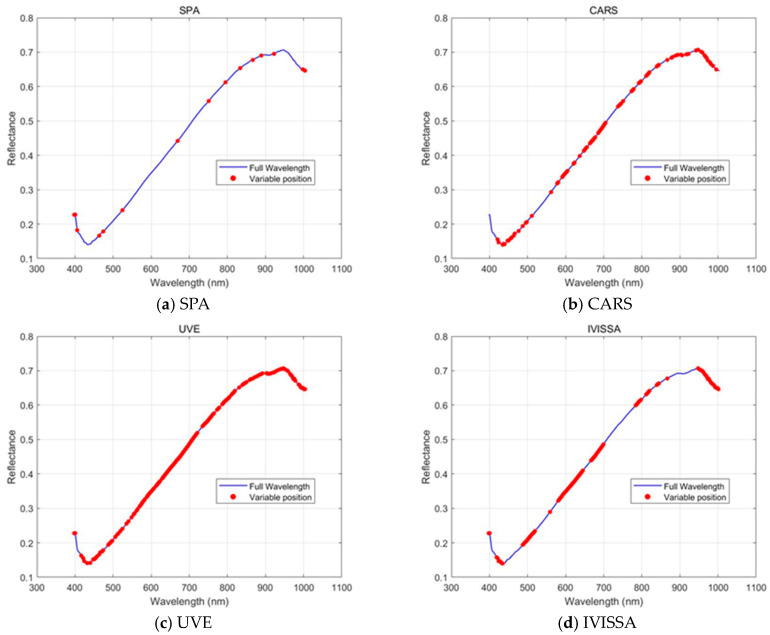
Distribution of selected spectral wavelengths by different feature extraction methods: (**a**) SPA; (**b**) CARS; (**c**) UVE and (**d**) IVISSA.

Although modeling with the full hyperspectral spectrum yields strong classification performance, it is often hindered by the curse of dimensionality and high computational costs [37]. To address these challenges, feature wavelength selection methods are used to reduce data dimensionality while retaining the most informative spectral features. Figure 6 shows the feature wavelengths selected by four methods: SPA, CARS, UVE, and IVISSA. Each method selects a specific number of wavelengths from the full spectral range, with the selected wavelengths highlighted in red on the spectra. These methods vary significantly in the number of wavelengths selected, the spectral regions covered, and the distribution of selected wavelengths, affecting their ability to capture spectral information for classification.

A key difference among the methods is the level of dimensionality reduction achieved. SPA selects only 15 wavelengths, reducing data dimensionality by 93.30%. This substantial reduction results in a compact representation of the spectral data, improving computational efficiency, though it sacrifices detailed spectral information. In contrast, CARS and IVISSA select 86 and 95 wavelengths, respectively, reducing dimensionality by approximately 60%. These methods provide a more detailed spectral representation, enabling the model to retain more information from the original data while still reducing the number of variables. UVE, selecting 159 wavelengths, reduces dimensionality by only 29.02%, maintaining a relatively high number of features, which can be beneficial for capturing fine spectral details but may increase the risk of overfitting [38].

When examining the repetition of selected wavelengths, it is noteworthy that CARS, UVE, and IVISSA each select multiple wavelengths from the same regions, suggesting that certain parts of the spectrum, particularly around 400–440 nm, 490–520 nm, 580–700 nm, and 880–960 nm, are more relevant for classification. These regions are associated with important spectral features linked to surface-level traits and biochemical compositions in rice grains [39]. In contrast, SPA selects wavelengths more sparsely across the full spectral range, with fewer repetitions in certain key spectral regions. The concentration of selected wavelengths in the 400–700 nm range suggests that the methods prioritize capturing visible spectral features, which are essential for distinguishing rice varieties based on surface composition and pigmentation. These differences highlight the trade-off between dimensionality reduction and retaining sufficient spectral information, reflecting an inherent conflict between reducing data dimensions and the associated computational cost.

### 3.6. Feature Wavelengths Modeling

As shown in Table 2, SNV preprocessing demonstrates superior performance across all modeling algorithms, suggesting that it effectively enhances the quality of the hyperspectral data. Consequently, the feature wavelength selection methods discussed in this section are applied to the data after SNV preprocessing, ensuring that the data quality is optimized prior to feature extraction. Similarly to the full-spectrum model, 4-fold cross-validation is used on the training set to select the best parameters and assess the generalization ability of the models. Table 3 presents the performance of different combinations of feature extraction methods and modeling algorithms.

Compared with full-spectrum modeling, only IVISSA-PLS-DA shows a slight overall improvement, whereas the other models decline to varying degrees, with larger drops observed for the deep learning models. Despite this reduction, CARS-CNN-Transformer still achieves the highest test accuracy (95.33%) and F1-score (95.40%) across all feature selection settings. The decreases seen in deep models are driven by the loss of spectral continuity and complementary information across adjacent bands on which CNN-based and attention-based representations depend; removing many correlated wavelengths shortens the effective sequence for attention and weakens long-range dependency modeling, and discarding apparently redundant bands reduces the implicit regularization that supports generalization. Notably, SPA selects 15 feature wavelengths, which produces the largest degradation because such a sparse set cannot preserve the cross-band interactions required for robust discrimination [40]; by contrast, CARS selects 86 feature wavelengths, retaining more informative regions and therefore causing a smaller drop and the best overall accuracy when paired with the CNN-Transformer.

Different modeling algorithms require distinct feature extraction methods to achieve optimal performance [41]. For instance, PLS-DA benefits most from IVISSA, with a 3.02% increase in accuracy. In contrast, SVM and 1D-CNN perform best with UVE, maintaining high performance while reducing dimensionality by 29.02%. Similarly, CNN-Transformer performs well when paired with CARS, reducing data dimensionality by 61.61%, while retaining nearly the same level of classification performance. UVE’s ability to reduce dimensionality while maintaining good performance suggests that this method strikes a balance between dimensionality reduction and classification performance, retaining a sufficient number of important features. However, CARS, which reduces dimensionality by a greater amount, still achieves similar performance, demonstrating that a more significant reduction in data size does not necessarily result in a loss of classification accuracy [42]. This suggests that the key factor is not just the reduction in dimensionality, but whether the modeling algorithm can effectively learn from the most relevant wavelengths and features for classification [43].

**Figure 7:**
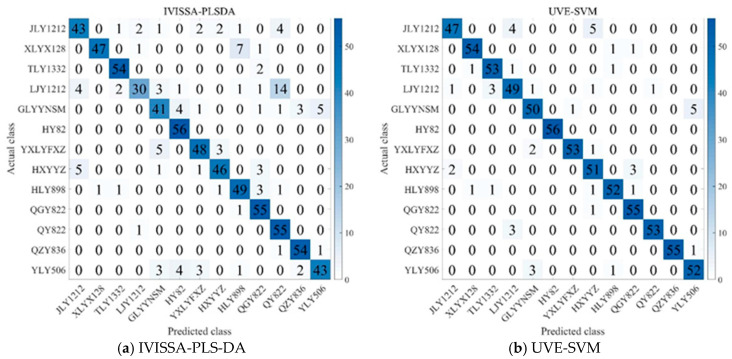

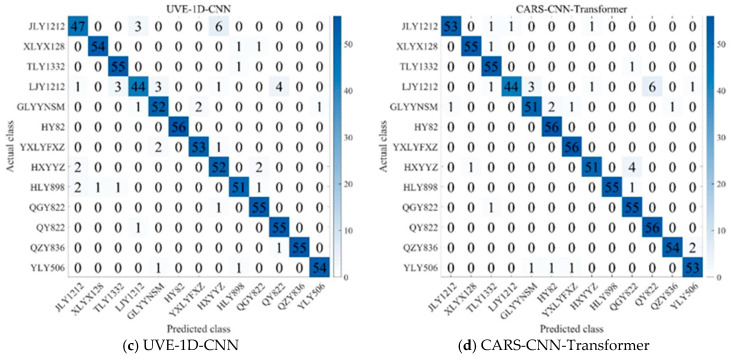
Confusion matrices of different modeling methods with optimal parameters: (**a**) IVISSA-PLS-DA; (**b**)UVE-SVM; (**c**) UVE-1D-CNN and (**d**) CARS-CNN-Transformer.

As shown in Figure 7, several key patterns emerge in the classification outcomes. JLY1212 is misclassified as TLY1332 and HXYYZ in a few cases, while LJY1212 is occasionally confused with TLY1332 and QY822. Similarly, HXYYZ is misclassified as JLY1212 and QY822 in some instances. In contrast, QY822 and TLY1332 are less frequently misclassified, suggesting that these varieties have relatively stable and distinctive spectral features, making them easier to distinguish from others. The fact that JLY1212, HXYYZ, and LJY1212 are more likely to be misclassified suggests that these varieties exhibit greater inter-variety differences, making it easier for the model to confuse them with other varieties [44]. HY82 demonstrates 100% classification accuracy across all models, but its recall is not always perfect, suggesting that while it is generally well identified, some models struggle to consistently classify it due to spectral variations or decision thresholds [45]. Additionally, varieties such as HLY898, QGY822, and QY822, despite sharing a common parental lineage, are consistently differentiated by the models, highlighting distinct spectral characteristics that allow for clear classification. These results highlight that subtle spectral differences, even among genetically related varieties, play a crucial role in classification, underscoring the importance of feature extraction methods and model tuning in achieving accurate rice variety classification.

### 3.7. SHAP Analysis

**Figure 8:**
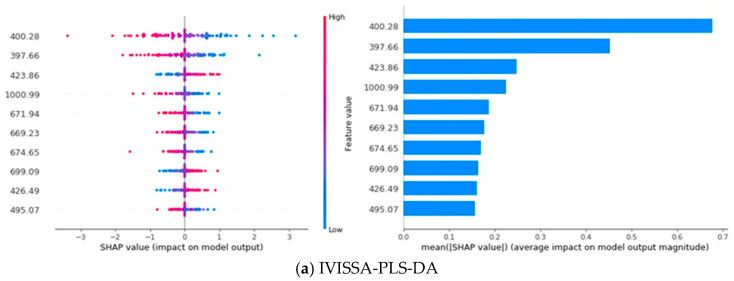

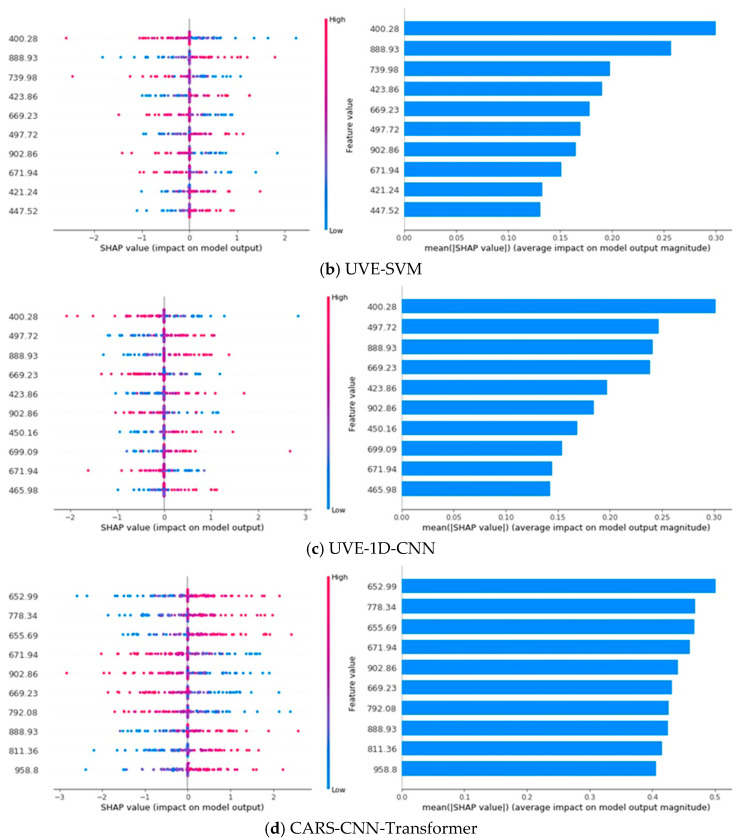
SHAP Analysis of Feature Importance for Different Models: (**a**) IVISSA-PLS-DA; (**b**)UVE-SVM; (**c**) UVE-1D-CNN and (**d**) CARS-CNN-Transformer.

In a SHAP plot, the horizontal axis represents the SHAP value, which reflects the impact of a feature on the model’s output; larger SHAP values indicate a greater influence on the prediction. The vertical axis represents the different features, with each point on the plot showing the SHAP value for a specific instance, where the color of the points indicates the feature’s value. The bar chart on the right displays the mean absolute SHAP value for each feature, representing the overall importance of each feature across all predictions. Features with longer bars have a greater average influence on the model’s output [46]. As shown in Figure 8, the SHAP analysis illustrates the contribution of different spectral features to the classification model’s performance. Specifically, the analysis focuses on the top 10 most influential spectral features for each model, emphasizing the wavelengths that have the greatest impact on classification decisions.

Notably, wavelengths such as 400.28 nm, 423.86 nm, 671.94 nm, 669.23 nm, 888.93 nm, and 902.86 nm consistently appear in the top ten features of at least three models, underscoring their importnce across different models. These wavelengths predominantly fall within the 400–440 nm, 580–700 nm, and 880–960 nm regions, aligning with the spectral features discussed in Figure 6, further validating their relevance for rice variety classification. Additionally, the SHAP analysis reveals a pattern where the impact of feature values depends on the wavelength [47]. For 400.28 nm, 671.94 nm, 669.23 nm, and 902.86 nm, the model’s output is more sensitive to lower feature values, with smaller values having a larger impact on the model’s prediction. Conversely, for 423.86 nm and 888.93 nm, higher feature values correspond to a greater impact on the model’s prediction, suggesting that these wavelengths behave differently in how they influence the model’s output.

Since each wavelength exhibits distinct spectral response patterns to specific grain constituents and chemical bonds, mapping high contribution wavelengths to their associated components helps identify the factors that most enable rice variety discrimination. The 400–440 nm region and the wavelength near 670 nm reflect pigment related absorption in the visible range, implying that differences in pigment accumulation contribute to class separation. Features between approximately 880 and 960 nm are sensitive to water and the macromolecular matrix, reflecting variation in moisture content and in the starch–protein composition captured by O–H, N–H, and C–H overtones. The 700–900 nm range is additionally modulated by endosperm microstructure that alters scattering. Taken together, pigment loading, moisture status, starch–protein matrix characteristics, and microstructural scattering constitute the principal factors underpinning varietal separability within the 400–1000 nm window.

Furthermore, the contribution distribution across models reveals distinct patterns. In PLS-DA and SVM, the first few key wavelengths dominate the model’s performance, accounting for the majority of the contribution, indicating that these models rely heavily on a few dominant features. In contrast, 1D-CNN exhibits a more balanced contribution distribution across features, implying that the model leverages a broader set of features for classification. CNN-Transformer, however, demonstrates a remarkable ability to learn from various features, with the contribution rate of the top 10 key wavelengths remaining nearly consistent, reflecting the model’s capacity to extract and utilize important spectral information from all features [48]. This consistency further underscores the powerful learning ability of CNN-Transformer, which enhances model performance through a diverse range of spectral features.

## 4. Conclusions

This study demonstrates the effectiveness of hyperspectral imaging combined with machine learning for classifying hybrid *indica* paddy rice grain varieties. The CARS-CNN-Transformer model achieved the best performance, with a classification accuracy of 95.33% and an F1-score of 95.40%. Key spectral regions, particularly in the 400–440 nm, 580–700 nm, and 880–960 nm ranges, were identified as crucial for distinguishing rice varieties. Notably, the CARS-CNN-Transformer model exhibited a balanced contribution across all key wavelengths, reflecting its powerful ability to extract useful features from diverse parts of the spectrum, thereby enhancing classification performance. This research underscores the potential of hyperspectral imaging and machine learning models for accurately classifying paddy rice grain varieties, offering insights for the development of automated systems for rice variety identification and quality control in agricultural practices.

However, this study has some limitations. First, it primarily focused on hybrid *indica* rice, and its applicability to other rice varieties, such as *japonica* rice, needs further evaluation. Second, despite controlling for factors such as geography, time, and field management, natural variations in the growth cycles of different rice varieties still result in sample differences. Finally, future research could explore the incorporation of additional features, such as texture characteristics and composite spectral indices, to further enhance model performance and generalizability.

## Figures and Tables

**Table 1:** Specific parameters of hyperspectral imaging system.

Parameters	Values
Wavelength range	397–1003 nm
Number of variables	224
Average interval	2.68 nm
Spectral resolution	5.5 nm
Image resolution	1024 × 1024 pixels
Sample conveying speed	7.5 mm/s
Camera exposure time	2 ms

**Table 2:** Comparison of different preprocessing and modeling methods based on full spectrum information.

Algorithms	Preprocessing Methods	Train Accuracy(%)	Test			
			Accuracy (%)	Precision (%)	Recall (%)	F1-Score (%)
PLS-DA	RAW	84.03	81.73	82.56	81.73	82.14
	SG	82.07	82.42	85.97	82.42	84.15
	BC	82.66	81.73	82.19	81.73	81.96
	SNV	81.83	82.28	85.91	82.28	84.06
	DT	80.70	80.77	82.37	80.77	81.56
	FD	79.12	79.53	80.73	79.53	80.13
SVM	RAW	86.13	84.62	85.37	84.62	84.99
	SG	85.92	84.48	85.23	84.48	84.85
	BC	83.38	80.22	81.15	80.22	80.68
	SNV	95.67	93.54	93.62	93.54	93.58
	DT	80.67	78.85	81.38	78.85	80.09
	FD	83.14	83.24	84.51	83.24	83.87
1D-CNN	RAW	92.79	84.20	84.42	84.20	84.31
	SG	92.55	83.93	84.09	83.93	84.01
	BC	92.79	85.58	85.94	85.58	85.76
	SNV	94.05	93.96	94.05	93.96	94.00
	DT	96.15	93.68	93.91	93.68	93.79
	FD	98.18	94.64	94.81	94.64	94.73
CNN-Transformer	RAW	95.02	95.05	95.19	95.05	95.12
	SG	95.33	93.82	93.96	93.82	93.89
	BC	94.78	95.33	95.42	95.33	95.38
	SNV	94.71	95.74	95.83	95.74	95.79
	DT	93.92	94.37	94.59	94.37	94.48
	FD	92.34	91.90	91.93	91.90	91.91

**Table 3:** Comparison of different combinations of feature extraction and modeling methods.

Algorithms	Feature Selection Methods	Train Accuracy (%)	Test			
			Accuracy (%)	Precision (%)	Recall (%)	F1-Score (%)
PLS-DA	SPA	74.28	74.59	78.16	74.59	76.33
	CARS	79.43	78.16	74.34	78.16	76.20
	UVE	84.34	82.83	83.36	82.83	83.10
	IVISSA	86.06	85.30	85.88	85.30	85.59
SVM	SPA	85.82	83.65	84.35	83.65	84.00
	CARS	91.90	90.52	90.82	90.52	90.67
	UVE	95.12	93.41	93.53	93.41	93.47
	IVISSA	92.79	92.03	92.11	92.03	92.07
1D-CNN	SPA	92.79	86.68	87.17	86.68	86.92
	CARS	95.67	91.76	91.79	91.76	91.78
	UVE	96.33	93.82	93.87	93.82	93.85
	IVISSA	95.36	92.31	92.41	92.31	92.36
CNN-Transformer	SPA	89.39	89.56	90.00	89.56	89.78
	CARS	95.09	95.33	95.47	95.33	95.40
	UVE	95.43	94.51	94.66	94.51	94.58
	IVISSA	93.03	93.96	94.00	93.96	93.98

## Data Availability

The data presented in this study are available on request from the corresponding author.

## Abbreviations

The following abbreviations are used in this manuscript:

PLS-DA	Partial Least Squares Discriminant Analysis
FD	First Derivative
T-SNE	T-Distributed Stochastic Neighbor Embedding
BOSS	Bootstrapping Soft Shrinkage
SVM	Support Vector Machine
ROI	Region of Interest
WT	Wavelet Transform
KNN	K-Nearest Neighbor
CNN	Convolutional Neural Network
SHAP	Shapley Additive Explanation
PCA	Principal Component Analysis
SG	Savitzky–Golay
BC	Baseline Correction
SNV	Standard Normalized Variate
DT	De-trending
SPA	Successive Projections Algorithm
CARS	Competitive Adaptive Reweighted Sampling
UVE	Uninformative Variable Elimination
IVISSA	Interval Variable Iterative Space Shrinkage Approach
1D-CNN	One-Dimensional Convolutional Neural Network
*TP*	True Positive
*TN*	True Negative
*FP*	False Positive
*FN*	False Negative
